# Letter Re: “Effect of Baseline Adjacent Segment Degeneration on Clinical Outcomes After Lumbar Fusion”

**DOI:** 10.1177/21925682251330264

**Published:** 2025-03-28

**Authors:** Leevi A. Toivonen, Heikki Mäntymäki, Lorin M. Benneker, Hannu Kautiainen, Marko H. Neva

**Affiliations:** 1 Department of Orthopedics and Traumatology, 60670 Tampere University Hospital and Tampere University, Tampere, Finland; 2 Department of Neurosurgery, 60670 Tampere University Hospital and Tampere University, Tampere, Finland; 3 Orthopedic Department, 388063 Sonnenhofspital, Bern, Switzerland; 4 Primary Health Care Unit, Kuopio University Hospital, Kuopio, Finland; 5 Folkhälsan Research Center, Helsinki, Finland

We want to thank you for your interest toward our article^1^ and excellent comments on it. Choosing the optimal surgical strategy continues to be an apical question for spine surgeons. Our findings favor the exclusion of end-stage degenerative adjacent segments without relevant stenosis from fusion constructs. Of course, clearly symptomatic, such as unstable or stenotic, levels need to be treated. Further prospective studies are needed to delineate which degrees of stenosis at collapsed adjacent levels will become a problem.

We believe, as you suggested, that sagittal alignment ranks higher in deformity surgery than in degenerative disorders. We believe adequate decompression and fusion of focalized instabilities is most relevant to the majority of patients with degenerative pathologies.

While the Pfirrmann classification would be simpler than the CIS for routine use, it lacks resolution to differentiate advanced degeneration, as you stated. In addition, we previously found that all the CIS sub-scores on their own were inadequate in this setting.^2^ We anticipate automatic prediction models combining extensive data and integrated to hospital systems will eliminate the inconvenience of complexities and improve patient care in the future.

We again thank you for valuable discussion.
